# Scalable diagnostic screening of mild cognitive impairment using AI dialogue agent

**DOI:** 10.1038/s41598-020-61994-0

**Published:** 2020-03-31

**Authors:** Fengyi Tang, Ikechukwu Uchendu, Fei Wang, Hiroko H. Dodge, Jiayu Zhou

**Affiliations:** 1 Department of Computer Science and Engineering, Michigan State University College of Engineering, East Lansing, USA; 2 College of Osteopathic Medicine, Michigan State University, East Lansing, USA; 3 Department of Healthcare Policy and Research, Weill Cornell Medical School, Cornell University, New York, USA; 4 Layton Aging and Alzheimer’s Disease Center, Department of Neurology, Oregon Health & Science University, Portland, USA

**Keywords:** Alzheimer's disease, Dementia, Predictive markers

## Abstract

The search for early biomarkers of mild cognitive impairment (MCI) has been central to the Alzheimer’s Disease (AD) and dementia research community in recent years. To identify MCI status at the earliest possible point, recent studies have shown that linguistic markers such as word choice, utterance and sentence structures can potentially serve as preclinical behavioral markers. Here we present an adaptive dialogue algorithm (an AI-enabled dialogue agent) to identify sequences of questions (a dialogue policy) that distinguish MCI from normal (NL) cognitive status. Our AI agent adapts its questioning strategy based on the user’s previous responses to reach an individualized conversational strategy per user. Because the AI agent is adaptive and scales favorably with additional data, our method provides a potential avenue for large-scale preclinical screening of neurocognitive decline as a new digital biomarker, as well as longitudinal tracking of aging patterns in the outpatient setting.

## Introduction

The search for early biomarkers of mild cognitive impairment (MCI) has been central to Alzheimer’s Disease (AD) and dementia research community in recent years. While there exists *in-vivo* biomarkers (e.g., beta amyloid and tau) that can serve as indicators of pathological progression toward AD, biomarker screenings are prohibitively expensive to scale if widely used among pre-symptomatic individuals in the outpatient setting^1^. Classically, the structural magnetic resonance imaging (MRI) modality has been shown to capture a set of physiologic markers in the AD pathological process^2,3^. However, the identification of MCI from normal aging (NL) is challenging with MRI due to the fact that structural changes in the brain at this phase are minor and hard to detect^4^. Although recent studies^5–7^ have shown that inferring structural connections among brain regions may provide promising results of MCI detection, they generally involve identifying structural changes that proceed from the point where clinical changes (i.e., physiologic changes from cognitive decline) have already occurred.

To identify MCI status at the earliest possible point, the feasibility of obtaining *preclinical markers* (i.e., before the onset of detectable physiologic changes) needs to be investigated. We note that in this study, we use the term “preclinical” to identify those who are likely to receive clinical diagnosis of Alzheimer’s Disease in the future, not necessarily based on the amyloid deposition, pathological tau, and neurodegeneration (ATN) biomarker-based framework presented in Jack *et al*.^8^ Specifically, we are interested in identifying those who are currently *clinically normal* but are on the trajectory to develop MCI in the near future. However, the identification of such developmental trajectories requires that we first identify a set of *invariant markers* that can distinguish MCI patients from normal aging crosssectionally.

Ideally, such markers should be inexpensive to obtain and scalable to applications outside of the clinical setting. Fortunately, recent studies have shown that simple *linguistic markers* such as word choice, phrasing (i.e., “utterance”) and short speech patterns possess predictive power in assessing MCI status in the elderly population^9^. Note that this is quite different from “speech markers” that involve auditory changes in pronunciations^10–12^ which reflect early symptomatic changes in speech generation. Behavior and social markers such as language, speech and conversational behaviors reflect cognitive changes that may precede physiological changes and offer a much more cost-effective option for preclinical MCI detection^13,14^, especially if they can be extracted from a non-clinical setting. However, extensive semi-structured conversation on the scale of several hours may be required to obtain reliable linguistic markers of MCI, as shown in the previous study^9^. In the current study, we are interested in not only identifying linguistic markers which distinguish MCI from those with normal cognition, but also in proposing methods that can compose *new sets of questions* capable of identifying MCI with only a few conversational turns with the participants.

We develop a prototype AI dialogue agent that conducts screening conversations with participants. Specifically, this AI agent must learn to ask the specific sequence of questions that are more likely to elicit responses containing linguistic markers that distinguish MCI form normal (NL) aging. We propose a reinforcement learning (RL)^15^ pipeline, along with a dialogue simulation environment^15–17^, to provide a training ground for the AI agent to explore over a range of semi-structured questions. The dialogue agent arrives at an optimal dialogue strategy – we call it a “policy”– for conducting adaptive conversations with users.

The proposed framework thus provides a potentially cost-effective and scalable way of screening the aging population for MCI-risk in an individualized manner. Our statistical learning approach leverages a principled way of minimizing generalization error^18^ which allows the agent to handle a diverse set of user conversational styles. Additionally, unlike classical supervised learning^18^, we incorporate a *feedback loop* between the RL and dialogue simulation module to allow rapid adaptation to unseen users without prior knowledge of their dialogue tendencies. In experiments, we demonstrate proof-of-concept results using cross-sectional data from a completed behavioral intervention trial. Results demonstrate that such an approach provides a potential avenue for longitudinal tracking of aging patterns through strategic and data-driven dialogue.

## Methods

### Study design and participants

We train and validate our AI dialogue agent based on transcribed data from a randomized controlled behavioral intervention NIH funded study (R01AG033581, ClinicalTrials.gov: NCT01571427) which was completed in 2014^19^. Briefly social isolation or lack of social interactions were found to be risk factors of dementia in epidemiological studies^20–22^. Therefore, this behavioral randomized controlled intervention trial aimed to examine whether increasing social interactions through video-chat conversations improve cognitive functions. User-friendly video-chat devices were created specifically for this project in order to reduce the effect derived from the stimulation of learning how to operate the device. The experimental group engaged in video-chat conversations with trained conversational staff for 30 minutes, 5 times per week for 6 weeks. Control group received only weekly 10 minutes phone check-in to monitor their social engagement activities and improve their retention. Participants were assessed by a full battery of neuropsychological tests used in all National Institute of Health (NIH)-funded Alzheimer’s Disease Centers in the United States (National Alzheimer’s Coordinating Center Uniform Data Set Version 2) at baseline and received clinical diagnosis by clinicians. To be eligible, the participants have to be at least 70 years old and free from frank dementia (mean age 80.43, 71% women, average years of education 15.7). Out of 83 subjects who completed the trial, we use the transcribed data from 41 subjects (14 MCI, 27 NL) in the current study who consented to have conversations transcribed and shared among researchers. There were no significant differences in age, gender, education and marital status between the group who consented to use their recorded conversation for this research study compared to those who did not. A subset of the total conversations (2.81 conversational episodes per participant) were transcribed and used for the current study. Basic *characteristics of participants*, *inclusion* and *exclusion criteria* are summarized in Supplementary Table 1.

### Conversation structure and preprocessing

The conversational transcripts were first processed into *utterances*, which are unstructured responses to questions provided by interviewers. The interview questions were generated from a pool of over 150 possible questions which are organized into the following categories: *transportation*, *childhood*, *cities*, *entertainment*, *family*, *personal preferences*, *gifts and celebrations, health problems, past jobs, spouse, social opinions, politics, comment about photographic images*, and *significant relationships*. During the interviews, the interviewer was allowed to adapt the specific wordings of questions to the participant. However, these behaviors are not accounted for in the current version of the AI conversation agent.

For the purposes of this study, we re-compiled the question list into 107 general questions which were ubiquitous across all conversations. For some of the questions, we *delexicalised* certain topic words such as “<*activity* > ”, “<*social topic* > ”, whereby specific nouns are replaced by contextual descriptors^23^. This is done to reduce the size of the question pool without sacrificing their contextual meaning. For participant responses, we did not remove “stopwords” (i.e. “uhh”, “hmm”), as their usage frequencies reflect some lexical properties of the user. A sample of the categorical interview questions is shown in Supplementary Table 2.

### Pretraining and fine-tuning skip-thought embeddings

The benefit of using language models such as word- and sentence-level embeddings is that these representations can be learned using datasets outside of our task. The rationale is that sentence representations have some invariant properties – so called *latent features* – that can be observed and learned from compositions of multiple types (texts, dialogues, speech, pose etc)^24^. We utilize a *pretraining* + *fine-tuning* framework^25^ whereby we initialize the language models used in our dialogue simulators with *Skip-thought* models pretrained on other corpuses. Specifically, we used pretrained *encoder* and *decoder* from Kiros *et al*.^26^, which is conditioned on over 74 million sentences from the *Bookcorpus* dataset. We then fine-tune the decoder based on dialogue responses from our data.

### MCI screening as a markov decision process

Reinforcement learning is a field within artificial intelligence (AI) research that aims to model strategic planning problems as Markov Decision Processes (MDPs)^15,27^. In this work, we model the trajectory of conversations in our dialogue problem as a MDP. Under such a framework, finding an optimal conversational policy involves decomposing the problem into a sequence of decisions, whereby the goal is to obtain the best action (i.e. question to ask) at each decision step based on the current *information state* (i.e. conversational state)^15,27^. We define the MDP for our problem $\{{\mathcal{S}},\,{\mathcal{A}},\,{\mathcal{T}},\,R,\,\gamma \}$ as follows:

- State space ${\mathcal{S}}$: a finite space of high-dimensional vectors representing the status of the conversation. This high-dimensional vector spaces captures sentence-level properties of responses from participants. In the original Asgari *et al*. work, “conversational states” are captured by summing LIWC word vectors for user responses^9^. Here, we encode conversational responses by training a sentence-level neural network that projects word embeddings to 4800-dimensional *Skip-Thought vector*s^26^. More details can about *Skip-Thought* (SKP) vectors can be found in Kiros *et al*.^26^. The benefit of SKP vectors is that they capture *sentence-level* features such as semantics, grammatical structure and various word choices. In our case, we use a *Skip-Thought* encoder to project user responses, which consist of sequences of words, into a *fixed length* vector of 4800 dimensions. As the conversation progresses, we use the transitional operator ${\mathcal{T}}$, described below, to encode the SKP vectors ${o}_{1},\ldots ,{o}_{t}$ of the user up to time $t$. The *latent state* of the transitional model represents the current conversational state, ${s}_{t}$.
- Action space ${\mathcal{A}}$: a set of discrete actions available at each state ${s}_{t}$ at time $t$. In our case, the action space is comprised of the pool of 107 categorical questions. At different stages of conversation, we designed censors over the available actions (i.e. no personal questions before introductions are made) to inject common knowledge about human conversations into agent’s decisions. We denote ${a}_{t}$ as the question output of the AI agent at time $t$, in response to the current conversational state $\,{s}_{t}$.
- Transition operator ${\mathcal{T}}$: an approximate description of the *dynamics* of conversations^27^. For our problem, ${\mathcal{T}}$ is a function which generates a response, i.e. a probability distribution over the next conversational state ${s}_{t+1}$, based on the current state ${s}_{t}$ and action ${a}_{t}$ and is learned directly from available data. We note here that the “conversational state” ${s}_{t}$ at time $t$ is different from the *SKP observation* of the user response at time $t$, which we denote ${o}_{t}$. Specifically, $\,{s}_{t}$ contains information from previous conversational turns while ${o}_{t}$ is only the encoded user response at time $t$. ${s}_{t}$ satisfies the property $p({s}_{t}\,|\,{s}_{1},\ldots ,{s}_{t})=p({s}_{t}|{s}_{t-1})$ (Markovian property) while the SKP observations do not.
- Formally, we design a recurrent neural network $f({o}_{1},\ldots ,\,{o}_{t},\,{a}_{t})$ to model the transition between ${s}_{t}$ to $\,{s}_{t+1}$, given the question ${a}_{t}$:
  $${s}_{0}=\,\max \,\{0,\,{o}_{0}^{T}{W}_{os}\}\,({\rm{Initialization}})$$
  $${z}_{t}=\,\max \,\{0,\,{o}_{t}^{T}{W}_{oz}+{s}_{t-1}^{T}{W}_{sz}\}\,({\rm{Update}}\,{\rm{Gate}})$$
  $${r}_{t}=\,\max \,\{0,\,{o}_{t}^{T}{W}_{or}+{s}_{t-1}^{T}{W}_{sr}\}\,({\rm{Reset}}\,{\rm{Gate}})$$
  $${h}_{t}=\,\tanh \,\{{o}_{t}^{T}{W}_{oh}+{({r}_{t}\odot {s}_{t-1})}^{T}{W}_{sr}\}\,({\rm{State}}\,{\rm{Update}})$$
  $${s}_{t+1}=(1-{z}_{t})\odot {h}_{t-1}+{z}_{t}\odot {h}_{t})\,({\rm{Transition}})$$
  $${o}_{t+1}={s}^{T}{W}_{y}+{b}_{y}\,({\rm{Output}})$$
  Here, the neural network $f({o}_{1:t};\,{a}_{t};\Theta )$ takes in SKP observations ${o}_{1},\ldots ,{o}_{t}$ and outputs the next SKP vector response ${o}_{t+1}$. We denote $\Theta =\{{{\rm{W}}}_{{\rm{oz}}},\,{{\rm{W}}}_{{\rm{os}}},\ldots ,{{\rm{W}}}_{{\rm{y}}},\,{{\rm{b}}}_{{\rm{y}}}\}$ as the set of weights parameterizing the recurrent neural network model. Initially, ${o}_{0}$ is the “greetings” state of the conversation, which is set to the SKP vector corresponding to a default greeting response (i.e., “hi”). As the conversation progresses, we update the *Update*, *Reset*, *State*, and *Transition* gates of *f* based on the observed SKP responses ${o}_{1},\ldots ,{o}_{t}$ and agent questions. Thus, we denote the repeated application of $f$ as *dialogue simulation* since the recurrent process of applying $f,\,{f}^{2},\ldots ,{f}^{t}$ generates a “trajectory” of conversational responses based on the agent questions ${a}_{1},\ldots ,{a}_{t}$. We train $f({o}_{1:t},\,{a}_{t};\Theta )$ based on the original dialogue data using the loss function:
  1 $$ {\mathcal L} (\Theta )=\frac{1}{2}\mathop{\sum }\limits_{t=0}^{T}{\Vert f({o}_{1:t},{a}_{t})-{\hat{o}}_{t+1}\Vert }^{2}+\lambda {\Vert \Theta \Vert }_{2}.$$
  ${\hat{o}}_{t+1}$ denotes the true SKP vector observed at time $t+1$ in the dialogue. This loss function trains the parameters $\Theta$ of the transition model $f$ by minimizing the distance between the predicted SKP vectors ($({o}_{t+1}=f({o}_{1:t},\,{a}_{t})$ and the actual SKP vector from the conversational data $({\hat{o}}_{t+1})$ across time, i.e., $1,\ldots ,T$. The $\lambda {\Theta }_{2}$ term is a regularization mechanism used to prevent overfitting on the training data when building the transition module during dialogue simulation. It is important to note that the transition rules differ among participants, each capturing different personal and topic preferences that cannot be captured by the sentence-level encoding of responses alone. These issues are addressed in the *Dialogue Simulation* section below.
- Reward function *R*: a set of rules which assigns a scalar value to each question based on response of the participant. In our MDP, we designed a *per-turn* penalty to limit the agent from conducting extensively long conversations. At the end of the conversation, we enforce a largely positive or largely negative reward, based on the prediction accuracy to ensure that the agent is asking questions which extract the relevant features pertaining to MCI status. More formally, we design the reward function as follows:
  $$R({s}_{t},{a}_{t})=\{\begin{array}{ll}-1-\tau , & non-terminal\,state\\ -50,\, & terminal\,state\,w/misclassification\\ +100,\, & terminal\,state\,w/correct\,status\end{array}$$
  Here, $\tau$ denotes a confidence threshold, which is defined as the probability of either the NL or MCI class having 0.65 or higher probability under the MCI classifier. The idea is to penalize the AI agent for each non-terminal conversational turn (i.e., questions that keep the conversation going), especially for questions that are asked while the AI agent is already “confident” in its prediction of the MCI status. This mechanism enforces the AI agent to be “efficient”, which is to end the conversation when it is confident in the data it has collected up to time *t*. This efficiency mechanism is counterbalanced by a cumulative reward based on accuracy: a large positive reward for producing sequences of questions leading to the correct MCI prediction, and a large negative reward for questions leading to incorrect predictions.
  We note here that for the terminal state reward signals, the reward function uses the *MCI classifier*, which utilizes ${\sum }_{t=1}^{T}{o}_{t}$ as input (rather than *s*_*t*_) to predict the MCI label at the end of the episode. This subtle difference distinguishes the fact that *s*_*t*_ is used by the RL system for state-tracking but the MCI classifier uses a separate set of features $({\sum }_{t=1}^{T}{o}_{t})$ to generate the reward signal.
- Discount factor $\gamma$: Since conversations can potentially “last forever”, a discount factor which serves to limit the contribution of future expected rewards; the damping of future signals serves to balance the tradeoff between asking enough questions to arrive at a confident MCI prediction and limiting the questions required to get there.

### Dialogue simulation

In the real-world setting, human users will have differences in conversational preference which may or may not be relevant to the underlying MCI status. The AI agent should thus be able to produce *individualized* sets of questions which can distinguish users with speech characteristics consistent with MCI compared to the NL control. For example, MCI participants use relational, filler and sentiment words with different frequencies compared to NL^9^, a pattern that can only be distilled from sequential dialogue turns with a given person.

We model individual differences in word choice as variance in transitional operators (i.e. ${{\mathcal{T}}}_{i}\in {\mathcal{T}}$) from our MDP. This was done by incrementally learning individualized transition operators ${T}_{1},\ldots ,{T}_{N}\in {\mathcal{T}}$, transferring a small subset of shared parameters from previous models ${T}_{1},\ldots ,{T}_{N-1}$ to initialize the learning of new ones ${T}_{N}$.

It should be noted that the AI agent does not have knowledge of the actual user transition dynamics as the dialogue simulators are part of the RL *environment*. Thus, in order to obtain states, the agent *estimates* the transition operators ${\mathcal{T}}$ by fitting the recurrent model on the observed SKP vectors using the loss function from Eq. 1. At each conversational turn, the agent only receives reward signals $R({s}_{t},\,{a}_{t})$ and observation of the response (*o*_*t*_) from the environment and adapts its internal model and policy network accordingly. Thus, for unseen participant in our dataset, the AI agent does not have prior knowledge of the linguistic tendencies of these participants when initiating the conversation. At test time, we only use the dialogue simulators to produce responses to the agent questions to compare to actual responses from the original dataset.

### Learning individualized conversational strategy with reinforcement learning

**Figure 1:**
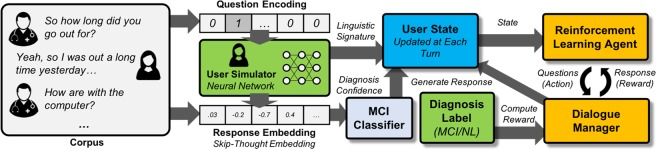
Feedback loop of the reinforcement learning environment for training the MCI diagnosis agent. The user simulator trained from the original dialogue corpus is used to generate simulated user response to new questions from the MCI diagnosis agent (i.e., the Reinforcement Learning Agent). At each conversational turn, the “user state” of the simulated patient is updated based on the questions asked by the MCI diagnosis agent. We designed a Dialogue Manager which produces a reward signal to the MCI diagnosis agent based on the quality of questions asked.

In reinforcement learning, there are several ways to train the agent to learn an optimal policy ${\pi }^{\ast }$ which solves the proposed MDP. A policy $\pi$ can be thought of as a strategy function which assigns a decision ${a}^{(t)}\in {\mathcal{A}}$ to each state ${s}_{t}\in {\mathcal{S}}$ at each time-step $t$^15^. While following a policy $\pi$, a *value function* ${Q}^{\pi }$ can be formulated to reflect the expected cumulative rewards for the agent while strictly adhering to the given strategy^15^. An *optimal* policy ${\pi }^{\ast }$ can thus be described as a strategy which traverses the state-action trajectories in a way that maximizes the expected cumulative rewards^15^. To solve for the optimal policy, we use *Deep Q-learning (DQN)*, a method which leverages properties of the *Q-*function to approximate a policy with provides the highest expected cumulative reward^28^. The advantage of this method is that a neural network is used to automatically learn the set of salient linguistic features from conversational responses which contribute to the accumulation of rewards and penalties, which in our case relate to the agent’s confidence over the MCI prediction accuracy, given the current conversational states, as well as the efficiency of conversational turns. An overview description of our entire dialogue system can be found in Fig. 1.

### Experimental setup and statistical analysis

To evaluate the conversational strategies discovered by the AI agent against the original dialogue conversations, we directly assess the area under receiver operating curve (AUC), sensitivity and specificity of MCI predictions based on the conversations generated by the AI agent and those generated by the interviewers from the original manuscript. We split the dataset into 65% training and 35% testing, and we perform 10 randomized shuffle splits and document the confidence interval (CI) across all generated test sets.

Due to the limited nature of the existing dialogue data, which cannot reflect responses of participants to novel sequence of questions, we used the dialogue simulators to sample approximate individualized responses to questions posed by the AI agent. Individualized conversation simulators are widely used in the field of natural language processing to evaluate the effectiveness of goal-oriented dialogue systems^17^. We account for the discrepancy between responses generated from dialogue simulation and real responses by *off-policy evaluation* in the proceeding sections.

In total, we deployed 41 dialogue simulators, corresponding to each participant in the dataset. During both the training and testing phases of reinforcement learning, the AI agent does not have access to the internal model of dialogue simulators. Instead, the simulator takes in questions from the AI agent and produces a s*imulated response* (i.e. sentences) which the AI agent uses to decide its actions. At testing phase, we use these simulators to estimate responses to the AI strategies $(\pi )$ for each participant. The responses to the human expert conversational questions $({\pi }_{expert})$ are simply the observed responses from the transcribed dialogue.

## Results

### Skip-thought representations of user responses are more predictive of MCI status

**Figure 2:**
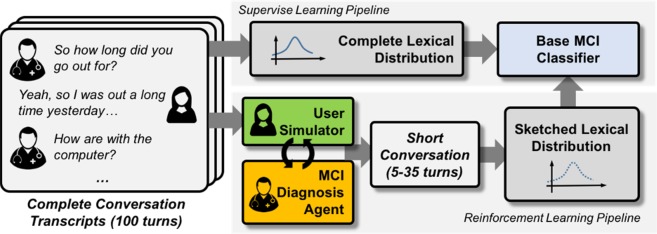
Overview of proposed algorithm for conversational generation and linguistic marker identification using a RL pipeline. Supervised learning pipeline denotes the classical approach by Asgari *et al*.^9^. Our approach is summarized in the RL pipeline and involves a feedback loop with the MCI diagnosis agent generating questions to new users for the purposes of predicting their MCI status using a trained ML classifier.

We first conduct MCI predictions based on the original set of transcribed conversational data from Oregon & Health Sciences University (OHSU)^19^. Details of the data can be found in *Materials and Methods* section below. Figure 2 illustrates the two main statistical learning approaches compared in this study.

Here, the classical ML approach presented in Asgari *et al*.^9^ for identifying MCI linguistic markers is denoted by the *supervised learning pipeline*. Under the supervised learning setting, all the conversational responses by a subject is compiled into a corpus of words. Responses are then converted into *word vectors*, where each dimension of the word vector is a {0,1} indicator of a distinguishable feature based on its grammatical usage, semantic meaning and various contextual identifiers. The specifics of the word vector dimensions are manually determined by linguists who produced the *Linguistic Inquiry and Word Count* (LIWC) mappings for more than 15,000 English words commonly used in NLP studies^29–31^. ML classifiers such as *support vector machines* (SVM) and feed-forward neural networks (MLP)^18^ are then applied on the word vectors to predict the {0,1} label of the MCI status. For the SVM classifier, we used the same settings as in Asgari *et al*.^9^ which showed that the SVM classifier with L1-regularization obtained the highest AUC, sensitivity and specificity for MCI prediction using the LIWC features. When using LIWC features (69 in total), we confirm that this is the case. Table 1 shows that our SVM implementation was able to achieve 0.712 AUC with confidence interval (CI) of (0.615–0.811) on 5 different randomized shuffle splits. Our SVM results are close to the reported 0.725 AUC in the original Asgari *et al*. paper^9^. We also see from Table 1 that a shallow neural network (MLP model with 2 layers, 512 units each) performed poorly using the LIWC features, with AUC of 0.689 (0.560–0.818). This is consistent with the fact that deep models are prone to overfitting, especially with small sample sizes and simple feature representations.

**Table 1:** Classification of MCI based on complete transcript vs. simulated conversations.

Model	AUC	F1-Score	Sensitivity	Specificity
SVM *w/ LIWC*	0.712 (0.612–0.811)	0.631 (0.500–0.761)	0.680 (0.476–0.886)	0.744 (0.563–0.922)
Supervised DL *w/ LIWC*	0.689 (0.560–0.818)	0.182 (0.055–0.370)	0.300 (0.010–0.758)	0.767 (0.364–0.970)
SVM *w/ SKP*	0.797 (0.719–0.879)	0.719 (0.591–0.846)	0.654 (0.473–0.835)	0.939 (0.855–1.0)
Supervised DL *w/ SKP*	0.811 (0.715–0.907)	0.642 (0.469–0.813)	0.600 (0.366–0.833)	0.911 (0.838–0.984)
RL (T = 5)	0.633 (0.535–0.703)	0.486 (0.288–0.680)	0.459 (0.280–0.630)	0.811 (0.661–0.936)
RL (T = 10)	0.741 (0.631–0.852)	0.590 (0.352–0.829)	0.560 (0.309–0.811)	0.922 (0.823–0.969)
RL (T = 15)	0.721 (0.618–0.827)	0.595 (0.399–0.790)	0.50 (0.327–0.713)	0.922 (0.856–0.987)
RL (T = 20)	0.809 (0.706–0.914)	0.726 (0.551–0.901)	0.620 (0.413–0.827)	0.988 (0.953–1.0)
RL (T = 30)	0.853 (0.796–0.914)	0.801 (0.733–0.880)	0.818 (0.678–0.958)	0.898 (0.828–0.969)
RL(T = 35)	0.859 (0.787–0.952)	0.808 (0.735–0.883)	0.818 (0.677–0.958)	0.911 (0.839–1.0)
Difference	0.0616 (−0.049–0.172)	0.089 (−0.078–0.259)	0.163 (−0.083–0.410)	−0.040 (−0.130–0.050)

Abbreviations: Parentheses denotes confidence interval (CI) for the metric. SVM denotes support vector machines classifier, and Supervised DL denotes 2-layer feed-forward neural network classifier. RL denotes reinforcement learning agent. For feature representation of corpus, LIWC is the original word-level embedding used in Asgari et al., 8. SKP denotes a 4800-dimensional Skip-Thought vector embedding was used to represent each conversational turn. A dialogue summary is obtained by averaging across all turn-based responses for each user. We then evaluate the performance of our RL-agent across 10 stratified shuffle splits. Each split uses 65% of data for training and 35% for testing.

By contrast, the inverse trend is observed when deep representations (*Skip-thought* vectors, denoted *SKP*) are used to represent the linguistic data. SKP representations differ from LIWC in that they are pretrained from other datasets prior to the construction of our user simulators (see Methodology for details). Under the SKP setting, conversational responses are projected into 4800-dimensional *Skip-Thought vector*s^26^ that capture *sentence-level* features such as semantics, grammatical structure and various word choices. A MCI classifiers are then trained to map *Skip-Thought* vector responses to the MCI labels by minimizing the binary cross-entropy loss between samples^18,26^. We see from Table 1 that the MLP model achieves an AUC value of 0.811 with CI of (0.715–0.907), which is comparable to the SVM classifier 0.797 (0.719–0.879). Thus, we show here that a neural network MCI predictor can produce slight improvements over SVM without drastically overfitting to the training data, despite higher model complexity^32^. This can be explained by the fact that SKP representations capture more complex, *sentence-level* features^33^, leading to a more expressive feature representation compared to the LIWC^9^, which only captures *word-level* features.

### AI-generated dialogues produce more predictive linguistic markers

The bottom part of Fig. 2, labeled *Reinforcement Learning (RL) Pipeline*, summarizes our approach, which consists of training an AI agent (i.e., MCI Diagnosis Agent) to generate questions with the user in a feedback loop in order to obtain the linguistic markers. Details of this approach will be outlined in the *Materials and Methods* section below. Table 1 further shows various AUC, sensitivity and specificity scores of conversations produced by the AI agent when given constraints on the length of conversations. For example, *RL(T* = *35)* means that the AI agent was given a maximum of 35 turns to complete the conversation with the simulated participant. This is because we noted that the average conversation in the original corpus lasted 35 turns. However, the dialogue corpus contained on average 2.8 30-minute conversations per participant, conducted within 6 weeks. The average number of total dialogue turns per person for supervised learning (i.e. SVM and supervised DL) is 107.5, which is more than 3 times the number of dialogue data needed by the AI-agent to produce comparable results.

The last line of Table 1 shows the *difference* in AUC, sensitivity and specificity scores per train-test split between *RL(T* = *2*0*)* and the *Supervised DL* model. The *RL(T* = *20)* agent generated 20 sequential questions and received 20 responses from the dialogue simulator for each test-set participant, which the AI agent has no prior conversational training data. The 20 responses are used by the 2-layer neural network to predict the MCI-status (*0* = *NL, 1* = *MCI*) for a given participant. In contrast, the *Supervised DL* model uses all the available dialogue responses for a given user to predict his or her MCI-status. We can see that the CI for their difference in AUC (−0.049–0.172), F1-score (−0.078–0.259), sensitivity (−0.083–0.410), and specificity (−0.130–0.050) all include 0.0. Beyond 20 turns, we see that the RL policy is able to achieve increasing performance in AUC scores. We quantify the impact of additional conversational turns in the proceeding sections.

### The AI policy adaptively finds the high-yield questions for unseen users

**Figure 3:**
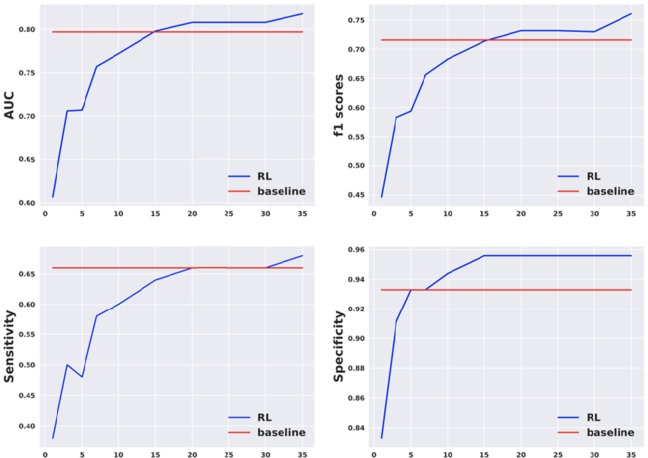
Conversational efficiency of AI agents. The x-axis represents the number of dialogue turns elapsed. The y-axis represents various performance metrics. Baseline refers to the performance of MCI classifier using all the responses generated from the original dataset. By contrast, RL refers to the performance of MCI classifier using responses generated by the user simulator, in response to the agent-generated questions at test time.

To further investigate the efficiency of our dialogue agent, we compared the rate of increase in the predictive power of linguistic markers as a function of AUC-gain per added conversational turn. In Fig. 3, we observe from the slope of the AUC-gain that the rate is fastest at the beginning, suggesting that the MCI diagnosis agent identifies and prioritizes the most relevant questions to assess MCI status early in the conversation. Perhaps most noteworthy is that at evaluation time, the MCI diagnosis agent has never seen the new subject and has to adapt its conversational strategy on-the-fly. As a result, we see that the AUC-gain curve is non-smooth: some prediction errors result from the fact that the new user behaves differently than the user simulation environment from which the diagnosis agent was trained in. However, the AUC-gain curves also demonstrate the capacity of the MCI diagnosis agent to self-correct its strategy in the face of measurement errors, in real-time, during new conversation.

Here, we define *conversational efficiency* as the number of dialogue turns required to achieve indistinguishable MCI-status prediction accuracy, as measured by overlapping confidence intervals of AUC, F1, sensitivity and specificity scores with expert interviewers *π*_*expert*_. Figure 3 illustrates that the *conversational efficiency* of the AI agent is 20, which means that the AI agent requires only 20 conversational turns to produce users’ responses whose C.I. measures are consistent with *π*_*expert*_ using the original dataset.

Furthermore, Table 2 shows the magnitude of incremental prediction improvement at 5, 10, 20, 30 and 35 turns between the prediction models. We bolded the rows in which the lower-bound on AUC confidence interval of the AI agent exceeds the upper-bound of the SVM confidence interval, indicating statistically significant improvement for the corresponding turn-adjusted predictions. Specifically, the AI agent produced statistically significant improvements at 10, 20, and 30 turns when compared with supervised learning methods. Interestingly, the neural network is also able to offer some improvement in the mean AUC scores across various turn restrictions, compared to the SVM, but it does not do so in a statistically significant way compared to the AI agents.

**Table 2:** MCI prediction of transcript and simulated conversations with turn restrictions.

Model (Turns)	AUC	F1-Score	Sensitivity	Specificity
SVM (T = 5)	0.493 (0.439–0.547)	0.169 (0.061–0.275)	0.12 (0.046–0.193)	0.860 (0.776–0.950)
SVM (T = 10)	0.550 (0.479–0.620)	0.275 (0.113–0.428)	0.200 (0.083–0.319)	0.900 (0.820–0.970)
SVM (T = 20)	0.624 (0.563–0.685)	0.405 (0.232–0.578)	0.360 (0.171–0.548)	0.888 (0.789–0.989)
SVM (T = 30)	0.633 (0.557–0.707)	0.424 (0.247–0.601)	0.320 (0.187–0.458)	0.944 (0.882–1.0)
SVM (T = 35)	0.714 (0.627–0.801)	0.576 (0.420–0.732)	0.440 (0.277–0.602)	0.968 (0.944–1.0)
Supervised DL (T = 5)	0.497 (0.392–0.603)	0.104 (0.015–0.182)	0.111 (0.091–0.129)	0.880 (0.812–0.980)
Supervised DL (T = 10)	0.527 (0.459–0.594)	0.278 (0.123–0.433)	0.200 (0.088–0.316)	0.933 (0.856–1.0)
Supervised DL (T = 20)	0.673 (0.588–0.758)	0.399 (0.212–0.583)	0.320 (0.139–0.500)	0.945 (0.888 - (1.0)
Supervised DL (T = 30)	0.720 (0.643–0.796)	0.477 (0.317–0.638)	0.360 (0.228–0.491)	0.955 (0.914–0.996)
Supervised DL (T = 35)	0.780 (0.695–0.864)	0.490 (0.327–0.654)	0.366 (0.229–0.500)	0.966 (0.928–1.0)
RL (T = 5)	0.633 (0.535–0.703)	0.486 (0.288–0.680)	0.459 (0.280–0.630)	0.811 (0.661–0.936)
**RL (T** = **10)**	**0.741 (0.631–0.852)**	**0.590 (0.352–0.829)**	**0.560 (0.309–0.811)**	**0.922 (0.823–0.969)**
**RL (T** = **20)**	**0.809 (0.706–0.914)**	**0.726 (0.551–0.901)**	**0.620 (0.413–0.827)**	**0.988 (0.953–1.0)**
**RL (T** = **30)**	**0.853 (0.796–0.914)**	**0.801 (0.733–0.880)**	**0.818 (0.678–0.958)**	**0.898 (0.828–0.969)**
RL(T = 35)	0.859 (0.787–0.952)	0.808 (0.735–0.883)	0.818 (0.677–0.958)	0.911 (0.839–1.0)

Abbreviations: SVM denotes support vector machines classifier, Supervised DL denotes 2-layer feedforward neural network, and RL denotes reinforcement learning agent. In all three cases, conversations were cut off at various turn lengths (T), and performance with the classifier was performed to obtain the AUC, F1, sensitivity and specificity scores. Confidence intervals were obtained on 10 randomized shuffle splits for all experiments.

### Quality of simulated responses depend on sentence lengths

In addition to quantitative metrics, we provide some concrete comparisons of dialogue simulations and the original dialogue corpus. Table 3 provides snapshots of 2 conversations, one with a verbose participant and one with a concise participant. In the left column, we see the original dialogue response to the questions posed by the interviewer. On the right column, we observe predicted response by the dialogue simulation based on maximum-likelihood estimation. It is notable that in short response (5–15 words), the dialogue simulation produces relatively stable responses both in terms of word choice as well as semantic meaning. On longer responses, as in the first user, we see that the dialogue simulator generates similar sentiment words as the original response, but the topical nouns and subject references may differ greatly.

**Table 3:** Comparison of AI and interviewer strategies using off-policy evaluation.

Policy	Avg. Reward/Turn	WIS Score	DR estim./turn	DR Score
RL (T = 35)	11.68 (2.06–21.35)	408.29 (72.41–744.17)	13.10 (12.91–13.35)	458.64 (452.40–464.87)
Expert Policy	2.62 (−7.28–12.51)	91.71 (−255.12 − 438.68)	10.82 (10.51–11.14)	379.07 (367.89–390.25)
Advantage	8.68 (7.16–10.13)	302.67 (250.78–354.58)	—	—

Weighted Importance Sampling (WIS) indicates off-policy evaluation of a given policy while sampling trajectories from the original dataset corpus25. For the expert policy, no importance weights are needed, and the cumulative rewards are used over entire conversational episodes. For the AI agent, a cut-off of 35 turns is again used to bound the length of off-policy trajectories. Average reward per turn is used to assess the average expected reward for the agent based on the reward function used to train the RL agent.

Evaluating the accuracy of dialogue simulation has long been a difficult problem in natural language research^17^. A major problem resides in the fact that word-by-word comparisons such as *perplexity* or BLEU score does not adequately capture changes in grammatical structure, sentiment and semantics between sentences^17^. For this reason, our analysis has focused mainly on *predictive ability* of the AI policy as a result of reinforcement learning under imperfect dialogue simulation.

### AI policy leads to questions with greater cumulative rewards during off-policy evaluation

To account for potential bias in our dialogue simulators, we also compared the AI conversational strategies ${\pi }^{\ast }$ against ${\pi }_{expert}$ directly over cumulative rewards on the original corpus conversations. Specifically, we deploy high-confidence off-policy evaluation (HCOPE)^34^ to weigh the reward difference between actions taken by the AI agent compared to the original actions in the corpus. In HCOPE, weighted importance sampling (WIS) is used to de-bias the rewards accumulated by the AI agent by bootstrapping the true policy values of the test set conversational trajectories^34^. In this setting, we evaluate both ${\pi }^{\ast }$ and ${\pi }_{expert}$ with the sample conversations from the original corpus at test time, scaling their episodic rewards by the WIS factor. Table 3 summarizes the *per-turn* reward and the *per-conversation* cumulative reward differences between the $\,{\pi }^{\ast }$ and $\,{\pi }_{expert}$. Here, we observe that $\,{\pi }^{\ast }$, as denoted by *RL(T* = *35)* accumulates on average 408.29 (72.41–744.17) while ${\pi }_{expert}$ accumulates on average 91.71 (−255.12–438.68) per conversation. Although the confidence intervals overlap, we see that both the cumulative advantage (250.78–354.58), and advantage per-turn (7.16–10.13) are non-zero in the C.I. interval, suggesting that ${\pi }^{\ast }$ produces significantly higher reward per action than the ${\pi }_{expert}$.

We note here that the interviewers in the original clinical trial were not instructed to optimize for the efficiency of conversation but rather to fill the entire 30 minutes of semi-structured conversation. However, we simply demonstrate here that a *goal-oriented* policy is possible – i.e., that reward signals and RL training regimes can be designed in a way that induce conversational strategies to survey the salient linguistic features using less time (as reflected in dialogue turns). By turning the original *supervised learning* problem into a *reinforcement learning* problem, we allow more efficient feature sensing strategies to be discovered.

## Discussion

In this work, we introduce a data-driven method for developing an automated and scalable diagnostic screening tool for efficient detection of early MCI status based on linguistic data. Traditionally, diagnostic predictions in the medical domain are modeled as *supervised learning* problems, whereby diagnostic labels provided by physician experts are used to guide the modeling process. By formulating the MCI screening problem as a Markov Decision Process (MDP)^15^, we transform the learning task into an *active sampling* process by which the AI agent participates both in the data mining process (interacting with a virtual user) as well as the prediction process (assessing the MCI status). The resulting AI agent obtains not only the ability to make diagnostic predictions, but also learns an efficient data-generating strategy for detecting the disease of interest in a natural setting.

In our experiments, we introduce a method for comparing the efficiency of AI dialogue strategy against that of the human interviewers. Specifically, we defined *conversational efficiency*, which quantifies the efficiency of different intervention strategies (AI-simulated vs. observed) based on the AUC-gains resulting from the different data provision strategies. Additionally, we also introduce an off-policy evaluation strategy which provides a lower-bound confidence on the expected performance of the AI policy compared to that of the human interviewers. Despite the imperfectness of the dialogue simulators, we illustrate a way to empirically assess the margin of error between the AI and human policies in a per-turn and per-conversation basis.

There are several limitations to our study. We note that MCI does not necessarily progress to AD or dementia and there are discrepancies between pathological burden and clinical diagnoses. However, this limitation is precisely the motivation behind our approach: cognitive prodrome states do not have clean signals that can distinguish “normal” from “pathologic” trajectories. Instead, we focus on detecting linguistic markers (text-based rather than acoustic, since changes in phoneme frequencies often suggest existing physiologic processes^11^), as the rise of heuristic search – through statistical machine learning or otherwise – can efficiently search through a potentially infinite space of dialogue sequences. Unlike classical statistical learning methods, our learning framework utilize a *feedback control* loop between a RL module and *simulation* to continuously improve language marker identification and enable *repeated predictions* through interaction with the patient.

Empirically, the most notable limitation is the size of the cohort and the setup of our study did not permit us to confirm the cognitive status of participants beyond cross-sectional analyses. We were also unable to confirm biomarkers (e.g., amyloid, tau) for our participants. It would be ideal if we could apply our methods to differentiate MCIs who were destined to develop dementia from those who remained normal using longitudinal follow-up. Groups with access to datasets with high-resolution and longitudinal biomarkers may be motivated to apply our proposed framework to (a) augment feature space of their models with our proposed linguistic marker acquisition protocol, (c) associate discovered linguistic markers with downstream biomarkers, and (b) test the reproducibility of our methods at scale.

In terms of statistical properties, all participants are in the 70+ age group and are from geographically similar areas. Although the p-values in Supplemental Table 1 show that such meta-data did not contribute in a statistically significant manner to MCI prediction, these factors inevitably introduce bias into our *simulation models*. We note that this is also the motivation behind using simulation during evaluation: ideally, we would like to conduct large-scale *active learning* during test time, involving large cohorts of patients. However, this would require the deployment of our framework in a real-world setting, a situation that prerequisites extensive efficacy in clinical trials. As a result, we present this study as a *proof-of-concept* to justify clinical trial and patient recruitment which would enable more powerful evaluation methods.

Additionally, there are several limitations with the construction and use of dialogue simulators in our experiments. Firstly, we note that punctuations were removed during the process, which lead to biased results during the SKP vector encodings, especially for longer sentences. However, since the original *Skip-thought* encoder was trained on the *Bookcorpus* dataset that featured 13.5 words-per-sentence on average, our performance on simulating short responses were relatively stable. Secondly, we restricted the set of questions on the *interviewer* side to a pool of 107 general questions (see Supplemental Table 2) that were observed across patients. In reality, the set of possible actions (i.e., questions) should be much richer than the one used in this preliminary study. One possible extension of this work is to investigate the feasibility of our hypothesis under a completely *open-dialogue* setting, whereby the dialogue agent and the user use both use *unstructured* questions and answers in conversation. Going from a pool of 107 questions to open dialogue requires a different question selection mechanism to be used in the RL pipeline, and the representation of questions will likely need to be adapted to achieve this. However, we expect that one can apply Guided Policy Search (GPS)^35^ to limit the deviation from the original set of questions.

Another limitation of our dialogue simulation is that it does not fully capture the difference between human-AI and human-human conversations in *real-world* conversation (i.e., the human-in-the-loop problem)^36^. One way we can alleviate this problem is to continuously improve the accuracy of our dialogue simulation. To fully capture the difference between human-AI and human-human conversations, a future direction of our study is to apply transfer learning to update our simulator models based on dialogue agents trained from other NLP datasets that are validated by *Wizard-of-Oz* evaluation^36,37^. Wizard-of-Oz evaluation is a technique in NLP research whereby the human interacts with a computer and does not know ahead of time whether the AI or another human will generate the questions for the proceeding conversation. The success of the dialogue agent in this case is measured by the degree to which the human user cannot distinguish between the agent’s questions from those of human questioners. Future studies may look to deploy *Wizard of Oz* evaluation on top of the RL pipeline to quantify the difference between human and AI delivery of questions^38^. Additionally, another promising direction include the immersion of our text-based approach with current state-of-the-art audio-based approaches^11,12^ as well as existing biomarkers. This step is beyond the scope of this prototype study, but it can improve the generalizability of our dialogue models and provide interpretability of the discovered features.

Finally, interpretability of the linguistic feature vectors (i.e., *skip-thought embeddings* of sentences) is an open problem in NLP. The original LIWC features in Asgari *et al*.^9^ provided a means of interpreting the linguistic feature vectors due to expert labeling of the latent dimensions (see Pennebaker *et al*.^30^ for details). However, the latent dimensions of *skip-thought* vectors are learned in an *unsupervised* manner (i.e., without expert labels). We are currently working to systematically study reliable interpretations of these latent features, but the interpretability of deep representations is a subfield of machine learning on its own right and outside the scope of the current study, which is to formulate an algorithm to discover *questions* which can elicit them.

The maturation of AI techniques in neuroscience is a process built in development cycles and clinical phases. We here introduce a novel framework for assessing the MCI status of aging populations that extends the application of ML methods beyond predictive modeling of disease processes. The proposed AI framework could provide a potentially cost-effective alternative to in-person interviews and may present a scalable way of screening for aging populations to distinguish normal aging from MCI-risk in an individualized manner. While still in a proof-of-concept phase, our results show a scalable and more robust version of our proposed framework provides an avenue for large-scale preclinical screening of neurocognitive decline through automated digital biomarker detection. If such a system is implemented at scale, longitudinal surveillance of dementia status can be greatly improved, potentially saving millions in outpatient costs and resource planning for the management of Alzheimer’s disease progression. More importantly, dialogue-based algorithms may present a step toward extending clinical care beyond the classical hospital and clinical settings.

### Ethics

Oregon Health & Science University Institutional Review Board approved the study protocol (protocol no. 5590), and all participants provided written informed consent. The project is listed in ClinicalTrials.gov (NCT01571427).

### Compliance

All methods were carried out in accordance with relevant guidelines and regulations.

## Supplementary information

Supplementary Information.

## Data Availability

The datasets generated during and/or analysed during the current study are not publicly available due to identifying information but are available from the corresponding author on reasonable request.
