# Supplementary material for: Scalable diagnostic screening of mild cognitive impairment using AI dialogue agent
Source: Sci Rep. 2020 Mar 31;10:5732. doi: 10.1038/s41598-020-61994-0 (PMC7109153; doi:10.1038/s41598-020-61994-0)
Supplement: Supplementary file 1 — Supplementary Information. [file 41598_2020_61994_MOESM1_ESM.docx]

**Supplementary Materials**

Table S1. Characteristics of participants.

Table S2. Sample Interview Questions.

**Supplementary Table 1*: Characteristics of participants.**

| **Variable** | **Unimpaired, *n* = 27** | **MCI, *n* = 14** | ***P*-value** |
| --- | --- | --- | --- |
| Age | 78.9 (5.5) | 83.4 (8.8) | .10 |
| Gender (% women) | 63 | 86 | .17 |
| Years of education | 16.6 (2.4) | 14.0 (2.6) | .003 |
| Marital status (% married) | 52.1 | 38.2 | .21 |

Inclusion Criteria:

1. Age 70 years or older

2. Clinical Dementia Rating (CDR) = 0 or 0.5

3. Sufficient vision and hearing to engage in conversation by personal computer system.

4. Sufficient English language skills to complete all testing.

5. General health status that will not interfere with ability to complete longitudinal study. Conditions that will likely lead to this problem are listed in the following in the study exclusions list.

Exclusion Criteria:

1. Plan to start taking new classes, traveling which requires more than two nights of stay away, or having significant social events such as a family wedding or a family reunion, during the scheduled prevention trial.

2. Diseases associated with dementia such as AD, ischemic vascular dementia, normal pressure hydrocephalus, or Parkinson's disease.

3. Significant disease of the central nervous system such as brain tumor, seizure disorder, subdural hematoma, cranial arteritis.

4. Alcohol or substance abuse diagnosis within the last 2 years

5. Comorbid major psychiatric disorders

6. Unstable or significantly symptomatic cardiovascular disease such as coronary artery disease with frequent angina, or congestive heart failure with shortness of breath at rest.

7. Active systemic cancer within 5 years of study entry.

8. Illness that requires >1 visit per month to a clinician.

9. Progressive vision loss (age-related macular degeneration already beginning to significantly degrade vision).

10. Need for oxygen supplementation for adequate function.

11. Medications:

a. Frequent use of high doses of analgesics

b. Sedative medications except for those used occasionally for sleep (use limited to no more than twice per week).

c. Applicable to CDR = 0.5 group only: subjects on unstable dosing of cholinesterase inhibitors (need to be stable dosing for 2 months).

*^*^Extracted from Table 2 of Dodge et al.* [*^17^*](https://paperpile.com/c/l26ble/zRQv)*. The details of the clinical trials data, neuropsychological and cognitive assessment protocols can be found at (*[*ClinicalTrials.gov*](http://clinicaltrials.gov/)*:* [*NCT01571427*](https://clinicaltrials.gov/ct2/show/NCT01571427)*).*

**Supplementary Table 2. Sample Interview Questions.**

| **Question Category** | **Question** |
| --- | --- |
| Activity | Did you go outside lately?  So what did you do today? |
| Social | Did you run into any familiar faces lately?  Where did you have dinner? |
| Picture | What do you see in this picture?  Where do you think this picture was taken? |
| Tech | How are you with the computer?  Did you use your computer lately? |
| Unspecified | <unspecified scheduling comment>  <unspecified picture comment> |
